# Clinical and genetic features of amyotrophic lateral sclerosis patients with *C9orf72* mutations

**DOI:** 10.1093/braincomms/fcad087

**Published:** 2023-03-21

**Authors:** Maximilian Wiesenfarth, Kornelia Günther, Kathrin Müller, Simon Witzel, Ulrike Weiland, Kristina Mayer, Christine Herrmann, David Brenner, Joachim Schuster, Axel Freischmidt, Dorothée Lulé, Thomas Meyer, Martin Regensburger, Torsten Grehl, Alexander Emmer, Susanne Petri, Julian Großkreutz, Annekathrin Rödiger, Robert Steinbach, Thomas Klopstock, Peter Reilich, Florian Schöberl, Joachim Wolf, Tim Hagenacker, Ute Weyen, Daniel Zeller, Albert C Ludolph, Johannes Dorst

**Affiliations:** Department of Neurology, Ulm University, 89081 Ulm, Germany; Department of Neurology, Ulm University, 89081 Ulm, Germany; Department of Neurology, Ulm University, 89081 Ulm, Germany; Department of Neurology, Ulm University, 89081 Ulm, Germany; Department of Neurology, Ulm University, 89081 Ulm, Germany; Department of Neurology, Ulm University, 89081 Ulm, Germany; Department of Neurology, Ulm University, 89081 Ulm, Germany; Department of Neurology, Ulm University, 89081 Ulm, Germany; Department of Neurology, Ulm University, 89081 Ulm, Germany; Department of Neurology, Ulm University, 89081 Ulm, Germany; Department of Neurology, Ulm University, 89081 Ulm, Germany; Department of Neurology, Center for ALS and other Motor Neuron Disorders, Charité—Universitätsmedizin Berlin, Corporate Member of Freie Universität Berlin, Humboldt-Universität zu Berlin, and Berlin Institute of Health, 13353 Berlin, Germany; Department of Molecular Neurology, Friedrich-Alexander-Universität Erlangen-Nürnberg (FAU), 91054 Erlangen, Germany; Alfried Krupp Hospital, Rüttenscheid, 45131 Essen, Germany; Department of Neurology, Halle University Hospital, 06120 Halle, Germany; Department of Neurology, Hannover Medical School, 30625 Hannover, Germany; Precision Neurology, University of Lübeck, 23538 Lübeck, Germany; Department of Neurology, Jena University Hospital, 07745 Jena, Germany; Department of Neurology, Jena University Hospital, 07745 Jena, Germany; Department of Neurology with Friedrich-Baur-Institute, University Hospital of Ludwig-Maximilians-University, 80336 München, Germany; German Centre for Neurodegenerative Diseases (DZNE) Site Munich, 81377 Munich, Germany; Munich Cluster for Systems Neurology (SyNergy), 81377 Munich, Germany; Department of Neurology with Friedrich-Baur-Institute, University Hospital of Ludwig-Maximilians-University, 80336 München, Germany; Department of Neurology with Friedrich-Baur-Institute, University Hospital of Ludwig-Maximilians-University, 80336 München, Germany; Department of Neurology, Diakonissen Hospital, 68163 Mannheim, Germany; Department of Neurology and Center for Translational Neuro and Behavioral Sciences (C-TNBS), University Hospital Essen, 45127 Essen, Germany; Department of Neurology, Ruhr-University Bochum, BG-Kliniken Bergmannsheil, 44789 Bochum, Germany; Department of Neurology, University of Würzburg, 97080 Würzburg, Germany; Department of Neurology, Ulm University, 89081 Ulm, Germany; German Centre for Neurodegenerative Diseases (DZNE) Site Ulm, 89081 Ulm, Germany; Department of Neurology, Ulm University, 89081 Ulm, Germany

**Keywords:** motor neuron disease

## Abstract

An expansion of the GGGGCC hexanucleotide in the non-coding region of *C9orf72* represents the most common cause of familial amyotrophic lateral sclerosis. The objective was to describe and analyse the clinical and genetic features of amyotrophic lateral sclerosis patients with *C9orf72* mutations in a large population. Between November 2011 and December 2020, clinical and genetic characteristics of *n* = 248 patients with amyotrophic lateral sclerosis carrying *C9orf72* mutations were collected from the clinical and scientific network of German motoneuron disease centres. Clinical parameters included age of onset, diagnostic delay, family history, neuropsychological examination, progression rate, phosphorylated neurofilament heavy chain levels in CSF and survival. The number of repeats was correlated with the clinical phenotype. The clinical phenotype was compared to *n* = 84 patients with *SOD1* mutations and *n* = 2178 sporadic patients without any known disease-related mutations. Patients with *C9orf72* featured an almost balanced sex ratio with 48.4% (*n* = 120) women and 51.6% (*n* = 128) men. The rate of 33.9% patients (*n* = 63) with bulbar onset was significantly higher compared to sporadic (23.4%, *P* = 0.002) and *SOD1* patients (3.1%, *P* < 0.001). Of note, 56.3% (*n* = 138) of *C9orf72,* but only 16.1% of *SOD1* patients reported a negative family history (*P* < 0.001). The GGGGCC hexanucleotide repeat length did not influence the clinical phenotypes. Age of onset (58.0, interquartile range 52.0–63.8) was later compared to *SOD1* (50.0, interquartile range 41.0–58.0; *P* < 0.001), but earlier compared to sporadic patients (61.0, interquartile range 52.0–69.0; *P* = 0.01). Median survival was shorter (38.0 months) compared to *SOD1* (198.0 months, hazard ratio 1.97, 95% confidence interval 1.34–2.88; *P* < 0.001) and sporadic patients (76.0 months, hazard ratio 2.34, 95% confidence interval 1.64–3.34; *P* < 0.001). Phosphorylated neurofilament heavy chain levels in CSF (2880, interquartile range 1632–4638 pg/ml) were higher compared to sporadic patients (1382, interquartile range 458–2839 pg/ml; *P* < 0.001). In neuropsychological screening, *C9orf72* patients displayed abnormal results in memory, verbal fluency and executive functions, showing generally worse performances compared to *SOD1* and sporadic patients and a higher share with suspected frontotemporal dementia. In summary, clinical features of patients with *C9orf72* mutations differ significantly from *SOD1* and sporadic patients. Specifically, they feature a more frequent bulbar onset, a higher share of female patients and shorter survival. Interestingly, we found a high proportion of patients with negative family history and no evidence of a relationship between repeat lengths and disease severity.

## Teaser

Wiesenfarth *et al*. report that amyotrophic lateral sclerosis patients with C9orf72 mutations differ significantly from sporadic patients and SOD1 gene carriers, including a higher share of bulbar onset, female patients, more severe neuropsychological deficits and shorter survival. No evidence of a relationship between repeat lengths and disease severity was found.

## Graphical Abstract

**Graphical Abstract:**
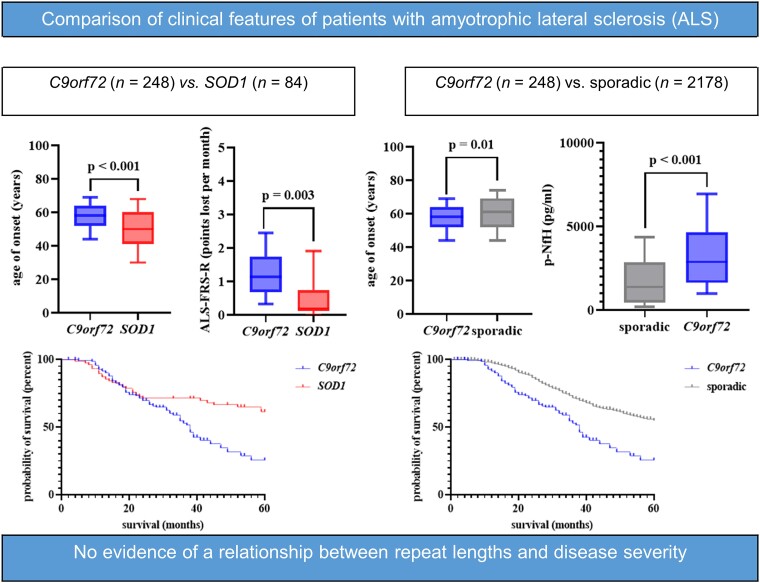

## Introduction

Amyotrophic lateral sclerosis is a progressive neurodegenerative disease which predominantly affects the upper and lower motor neurons. It is characterized by progressive muscle weakness and a severely reduced life expectancy of about 2–5 years after diagnosis.^1^ The worldwide all-age prevalence of motor neuron diseases is 4.5/100,000, the all-age incidence 0.78/100 000 people-years.^2^ In about 15% of cases, a causative gene mutation can be detected.^1^ An expansion of the GGGGCC hexanucleotide in the non-coding region of *C9orf72* represents the most common cause of familial amyotrophic lateral sclerosis.^3^ In previous studies, the share of causative *C9orf72* mutations ranged from 7.8^4^ to 41%^5^ of all patients with a positive family history and explained 5%^4^ of seemingly sporadic cases, depending on the composition of patients studied. *C9orf72* expression was found in the brain, spinal cord, myeloid cells and in several other tissues. *C9orf72* is believed to be involved in the regulation of autophagy, vesicular transport and inflammation.^6^ As a possible pathomechanism, both a loss of function and a toxic gain of function by accumulation of RNA foci or dipeptide repeat proteins are discussed.^7,8^ Of note, *C9orf72* mutations are not only the most common known genetic cause for the development of amyotrophic lateral sclerosis, but also for frontotemporal dementia (FTD) in Europe and North America.^3,7,9^

Riluzol^10^ and (in some countries) edaravone^11^ and sodium phenylbutyrate and taurursodiol^12^ are currently the only approved drugs for amyotrophic lateral sclerosis with limited effects. In genetically determined amyotrophic lateral sclerosis forms, the application of antisense oligonucleotides is generally regarded as a promising potential treatment option which is currently evaluated in clinical studies for various genetic mutations.^13^ In order to evaluate therapeutic effects of *C9orf72*-specific approaches, it is essential to characterize the clinical phenotypes and disease courses of affected patients as accurately as possible. In addition, it is useful to compare these patients to sporadic patients without amyotrophic lateral sclerosis-related gene mutations and with patients carrying other genetic mutations such as *SOD1* in order to identify mutation-specific characteristics.

For this purpose, we analysed clinical parameters including age of onset, body mass index (BMI), diagnostic delay, family history, gender distribution, site of onset, neuropsychological status, progression rate and survival in *n* = 248 patients with amyotrophic lateral sclerosis carrying mutations in the *C9orf72* gene and compared them to *n* = 84 amyotrophic lateral sclerosis patients with *SOD1* mutations and *n* = 2178 sporadic amyotrophic lateral sclerosis patients without any known amyotrophic lateral sclerosis-related genetic mutations.

## Materials and methods

### Subjects

Patients were enrolled from the database of the MND-net, a clinical and scientific network of 21 German motoneuron disease centres. We identified *n* = 248 patients from 12 centres (University of Ulm, Charité Berlin, Bergmannsheil University Hospital Bochum, University of Erlangen, Alfried Krupp Hospital Essen, University Medicine Essen, Halle University Hospital, Hannover Medical School, Jena University Hospital, Ludwig Maximilians University Munich, Diakonissen Hospital Mannheim and University of Würzburg) who were diagnosed with definite, probable or possible amyotrophic lateral sclerosis according to revised El Escorial criteria between 2012 and 2020 and who were positively tested for a GGGGCC hexanucleotide expansion in the *C9orf72* gene. Consistent with the literature,^9^ repeat expansions with more than 20 repeats were assumed to be pathogenic, whereas expansions with 20 repeats or less were assumed to be wild-type alleles.

All patients provided written informed consent. The study was approved by the local institutional ethics committees (application number 19/12).

### Outcomes

Demographic and clinical data included sex, date of birth, disease onset (defined as occurrence of first paresis), date of diagnosis, date of last follow-up, date of death, family history of amyotrophic lateral sclerosis, site of onset, BMI, Amyotrophic Lateral Sclerosis Functional Rating Scale-Revised (ALSFRS-R), Edinburgh Cognitive and Behavioural Amyotrophic Lateral Sclerosis Screen (ECAS) and phosphorylated neurofilament heavy chain (p-NfH) levels in CSF, if available. Diagnostic delay was defined as the interval between disease onset and diagnosis. Disease progression rate was defined as loss of ALSFRS-R score per month as calculated by the formula (48-ALSFRS-R at last visit) divided by months between onset and last visit.

Demographic and clinical data of the comparator groups (*n* = 84 patients with *SOD1* mutations and *n* = 2178 sporadic patients without evidence of a causative genetic mutation) were likewise enrolled from the MND-net database analogous to patients carrying *C9orf72* mutations. Patients were generally seen in three to six monthly time intervals in the outpatient clinics of the respective centres, and all available data from each patient were considered.

### DNA sequencing and analysis

Amyotrophic lateral sclerosis patients with positive family history for amyotrophic lateral sclerosis and sporadic patients with young age of onset as well as sporadic patients who agreed to genetic testing via the German MND network for scientific purposes were genetically tested. Due to the emerging promising gene therapies, all sporadic and familial patients who agreed to genetic testing have been routinely tested since 2020. Genetic testing was performed at the Institute of Human Genetics of Ulm University.

DNA was extracted from blood leucocytes. Analysis of the *C9orf72* repeat length was performed by fragment length analysis and repeat-primed PCR.^3,9^ Electrophoresis was performed on an ABI PRISM® 3130 Genetic Analyzer (Life Technologies, Foster City, California, USA). The data were analysed using the Peak Scanner software (Applied Biosystems, Waltham, Massachusetts, USA). Samples with a sawtooth pattern in the repeat-primed PCR were further analysed using Southern blot.^14^ Screening for *SOD1* was done by Sanger sequencing for all coding exons and flanking 50 bps of *SOD1*.

### Neuropsychological examination

For cognitive testing, the ECAS with age- and education-stratified cut-offs was performed in a subgroup of *n* = 80 patients. ECAS includes amyotrophic lateral sclerosis-specific domains such as executive functions, verbal fluency and language, but also non-amyotrophic lateral sclerosis-specific domains such as memory and visuospatial orientation. Follow-up examinations were available for *n* = 16 patients.

### Statistical analysis

For descriptive statistics, median (IQR) or mean ± SD are given as appropriate. For group comparisons, the chi-square test was applied for nominal variables. Unpaired Student’s *t*-test was used analysing continuous variables and non-parametric Mann-Whitney U-test for non-normally distributed variables. One-way ANOVA analysis with *post hoc* Tukey’s test was performed for three group comparisons. Kaplan–Meier curves and log-rank test were applied to determine the effect of demographic or clinical parameters on survival. A *P*-value of ≤ 0.05 was regarded as statistically significant.

### Data availability

The data that support the findings of this study are available from the corresponding author, upon reasonable request.

## Results

Overall, *n* = 248 patients with amyotrophic lateral sclerosis carrying a mutation in the *C9orf72* gene were identified and included in the study. Demographic and clinical characteristics of patients with *C9orf72* mutations as well as the comparator groups consisting of *n* = 84 patients with *SOD1* mutations and *n* = 2178 patients without evidence of a causative mutation are shown in Table 1.

**Table 1:** Demographic and clinical characteristics of the study population

	C9orf72 (*n* = 248)	Sporadic (*n* = 2178)	SOD1 (*n* = 84)
**Age of onset** (median, IQR)	58.0 (52.0–63.8) (*n* = 220)	61.0 (52.0–69.0) (*n* = 2136)	50.0 (41.0–58.0) (*n* = 79)
**Sex**			
Male	51.6% (*n* = 128)	58.7% (*n* = 1264)	56.6% (*n* = 47)
Female	48.4% (*n* = 120)	41.4% (*n* = 891)	43.4% (*n* = 36)
**Onset**			
Spinal	66.1% (*n* = 123)	76.6% (*n* = 1484)	96.9% (*n* = 62)
Bulbar	33.9% (*n* = 63)	23.4% (*n* = 453)	3.1% (*n* = 2)
**Type**			
Sporadic	56.3% (*n* = 138)		16.1% (*n* = 13)
Familial	43.7% (*n* = 107)		84.0% (*n* = 68)
**ALSFRS-R^a^** (1st visit) (median, IQR)	39.0 (33.0–42.0) (*n* = 210)	39.0 (32.3–43.0) (*n* = 2012)	39.5 (31.0–44.0) (*n* = 60)
**Progression rate** (median, IQR) (1st to last visit)	1.1 (0.7–1.7) (*n* = 105)	0.8 (0.3–1.5) (*n* = 920)	0.2 (0.1–0.7) (*n* = 32)
**BMI** ^b^ **in kg/m²** (median, IQR)	24.1 (21.5–27.1) (*n* = 210)	24.3 (21.8–27.0) (*n* = 1896)	25.9 (22.6–28.4) (*n* = 37)
**Diagnostic delay in months** (median, IQR)	8.0 (4.0–14.0) (*n* = 189)	10.0 (6.0–17.0) (*n* = 1971)	11.0 (5.5–34.0) (*n* = 45)
**Survival in months** (median, HR, 95% CI)	38.0 (*n* = 144)	76.0 (0.43, 0.30–0.60) (*n* = 2106)	198.0 (0.51, 0.35–0.75) (*n* = 78)
	vs. *SOD1* (1.97, 1.34–2.88)		
	vs. sporadic (2.34, 1.64–3.33)		

a ALSFRS-R, Amyotrophic Lateral Sclerosis Functional Rating Scale-Revised; ^b^BMI, body mass index.

We found an approximately equal sex distribution with 48.4% (*n* = 120) women and 51.6% (*n* = 128) men in the *C9orf72* group. The median age of disease onset was 58.0 (IQR 52.0–63.8) years. Only 43.7% of patients reported on other cases of amyotrophic lateral sclerosis in their family, whereas 56.3% had no evidence of a positive family history and were therefore classified as sporadic cases. The onset of symptoms was spinal in 66.1% and bulbar in 33.9%. The median ALSFRS-R was 39.0 (IQR 33.0–42.0) at the time of diagnosis and decreased with a median progression rate of 1.1 points per month (IQR 0.7–1.7) during the time interval between the first and last visit. The median BMI at first visit was 24.1 kg/m² (IQR 21.5–27.1). We found a median diagnostic delay of 8.0 months (IQR 4.0–14.0). Median survival from onset was only 38.0 months.

### C9orf72 versus SOD1

**Figure 1:**
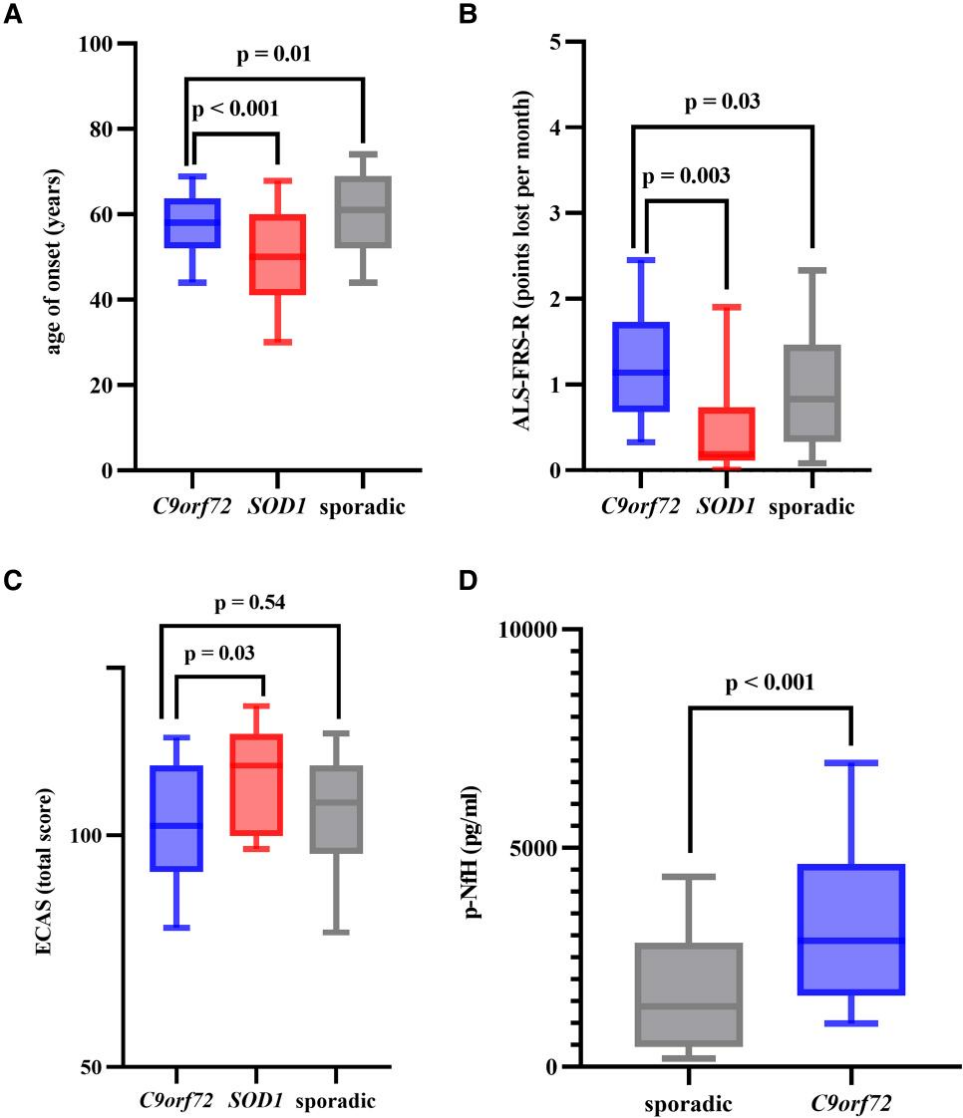
**Boxplots show median (IQR; 10–90 percentile) of clinical characteristics in C9*orf72* mutation carriers versus *SOD1* mutation carriers versus sporadic patients.** (**A**) Age of onset. (**B**) ALSFRS-R. (**C**) ECAS overall score. (**D**) p-NfH blood levels (only a few data of SOD1 available). Experimental units *n* = number. (**A**) *C9orf72 n* = 220, *SOD 1 n* = 79, sporadic *n* = 2136, *P* < 0.0001 in ANOVA, *C9orf72* versus *SOD1 P* < 0.0001 and *C9orf72* versus sporadic *P* = 0.0103 in *post hoc* Tukey’s test. (**B**) *C9orf72 n* = 210, *SOD 1 n* = 60, sporadic *n* = 2012, *P* = 0.0023 in ANOVA, *C9orf72* versus *SOD1 P* = 0.0029 and *C9orf72* versus sporadic *P* = 0.0305 in *post hoc* Tukey’s test. (**C**) *C9orf72 n* = 80, *SOD 1 n* = 20, sporadic *n* = 753, *P* = 0.0400 in ANOVA, *C9orf72* versus *SOD1 P* = 0.0301 and *C9orf72* versus sporadic *P* = 0.5390 in *post hoc* Tukey’s test. (**D**) *C9orf72 n* = 43, sporadic *n* = 339, *P* < 0.0001. One-way ANOVA-analysis with *post hoc* Tukey’s test was performed for three group comparisons. Unpaired Student’s *t*-test was used for two group comparison. A *P*-value of ≤ 0.05 was regarded as statistically significant. ALSFRS-R, Amyotrophic Lateral Sclerosis Functional Rating Scale-Revised; *C9orf72*, chromosome 9 open reading frame 72; ECAS, Edinburgh Cognitive and Behavioural Amyotrophic Lateral Sclerosis Screen; p-NfH, phosphorylated neurofilament heavy chain; *SOD1*, superoxide dismutase 1.

**Figure 2:**
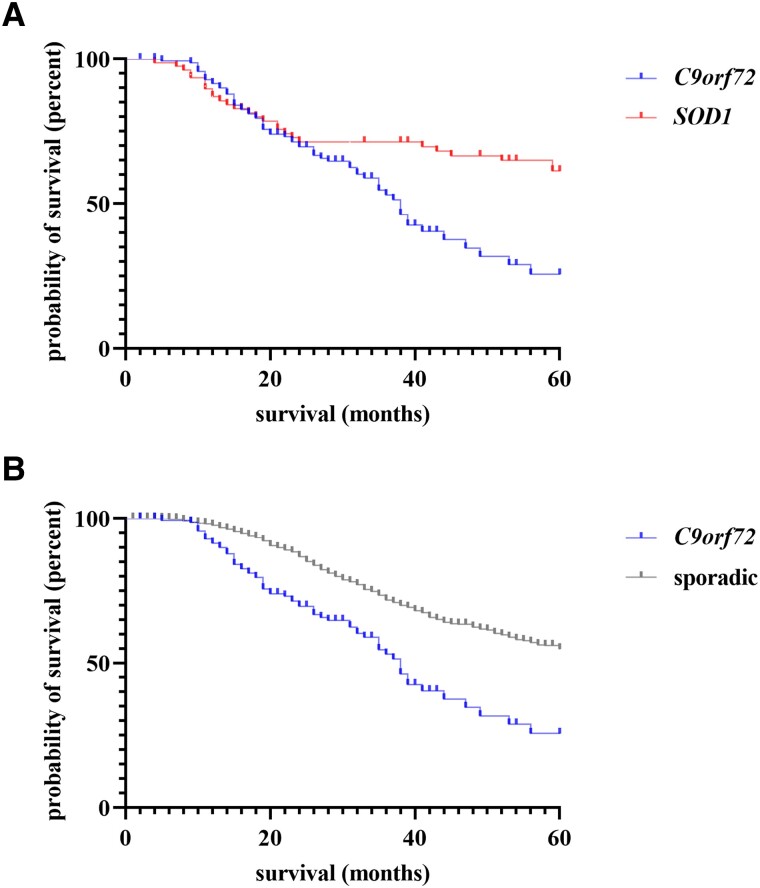
**Kaplan–Meier curves for survival in C9*orf72* mutation carriers versus *SOD1* mutations carriers versus sporadic patients.** Experimental units *n* = number. (**A**) *C9orf72 n* = 144, *SOD 1 n* = 78, *P* = 0.0002. (**B**) *C9orf72 n* = 144, sporadic *n* = 2106, *P* < 0.0001. Kaplan–Meier curves and log-rank test were applied to determine the effect of demographic or clinical parameters on survival. A *P*-value of ≤ 0.05 was regarded as statistically significant. *C9orf72*, chromosome 9 open reading frame 72; *SOD1*, superoxide dismutase 1.

The most common mutations in our group of patients with *SOD1* mutations (p.Arg116Gly *n* = 26, p.Asp91Ala *n* = 11, and p.Leu145Phe *n* = 6) are generally associated with a comparatively benign course of disease (Supplementary Table 1) which has to be kept in mind when interpreting the following results.^15^ Compared to amyotrophic lateral sclerosis patients with *SOD1* mutations (*n* = 84), the median age of onset of *C9orf72* mutation carriers was 8.0 (*SOD1*: 50.0 years, IQR 41.0–58.0) years later (*P* < 0.001; Fig. 1A). As opposed to *C9orf72*, the vast majority of *SOD1* patients (84.0%) reported a positive family history and featured a spinal onset (96.9%; *P* < 0.001). On their first visit, the median ALSFRS-R of *SOD1* patients (39.5, IQR 31.0–44.0) was similar to *C9orf72* (39.0, IQR 33.0–42.0) (*P* = 0.42). However, during the further course of the disease, their progression rate was significantly slower with a median decrease of 0.2 points (IQR 0.1–0.7) of ALSFRS-R lost per month (*P* = 0.003; Fig. 1B). The median diagnostic delay in *SOD1* patients was longer (11.0 months, IQR 5.5–34.0; *P* < 0.001). Median survival from onset was 198.0 months and therefore significantly longer compared to *C9orf72* (HR 0.51, 95% CI 0.35–0.75; *P* < 0.001; Fig. 2A).

### *C9orf72* versus sporadic amyotrophic lateral sclerosis

We likewise identified significant differences comparing patients with *C9orf72* mutations with sporadic amyotrophic lateral sclerosis patients (*n* = 2178), i.e. patients without a positive family history and without any known causative genetic mutation. In sporadic patients, we found a higher share of male patients (58.7%) compared to *C9orf72* (*P* = 0.03). The median onset of the disease was 61.0 years (IQR 52.0–69.0; Fig. 1A) and therefore 3.0 years later compared to *C9orf72* (*P* = 0.01). We found a bulbar onset in 23.4% (*n* = 453) of sporadic patients which was lower compared to *C9orf72* (*P* = 0.002). The median ALSFRS-R at the time of diagnosis was similar to *C9orf72* (39.0, IQR 32.3–43.0; *P* = 0.26), but decreased more slowly with a median progression rate of 0.8 points of ALSFRS-R (IQR 0.3–1.5; Fig. 1B) lost per month (*P* = 0.03). The median diagnostic delay in sporadic patients was 10.0 months (IQR 6.0–17.0) and therefore longer if compared to *C9orf72* (*P* = 0.02). The median survival from onset was longer with 76.0 months (HR 0.43, 95% CI 0.30–0.60; *P* < 0.001; Fig. 2B).

Median p-NfH concentrations in CSF were higher in *C9orf72* (2880 pg/ml; IQR 1632–4638 pg/ml; *n* = 43) compared to sporadic patients (1382, IQR 458–2839 pg/ml; *n* = 339; *P* < 0.001; Fig. 1D). A comparison to *SOD1* was omitted as only a few data were available.

### Effect of repeat lengths

We also analysed the relationship between clinical parameters and the number of GGGGCC hexanucleotide repeats. There were no significant differences with regard to sex distribution, site of onset, family history, BMI, ALSFRS-R, progression rate, diagnostic delay, p-NfH concentrations in CSF and survival between patients with >2000 (*n* = 78) and <2000 (*n* = 107) repeats as well as between patients with >2200 (*n* = 36) and <1500 (*n* = 31) repeats (Table 2). Counterintuitively, we found that the onset of the disease was earlier in the patients with lower repeat lengths (<2000 versus > 2000; *P* = 0.01 and <1500 versus > 2200; *P* = 0.02).

**Table 2:** Clinical features of patients with high versus low repeat lengths

	*C9orf72* < 1500 repeats (*n* = 31)	*C9orf72* > 2200 repeats (*n* = 36)	*P*-value
**Age of onset** (median, IQR)	56.0 (50.0–61.7) (*n* = 28)	62.0 (55.0–66.0) (*n* = 31)	**0**.**02**
**Sex**			0.99
Male	54.8% (*n* = 17)	55.6% (*n* = 20)	
Female	45.2% (*n* = 14)	44.4% (*n* = 16)	
**Onset**			0.99
Spinal	70.8% (*n* = 17)	74.1% (*n* = 20)	
Bulbar	29.2% (*n* = 7)	25.9% (*n* = 7)	
**ALSFRS-R^a^** (1st visit) (median, IQR)	41.0 (37.5–44.0) (*n* = 26)	39.5 (34.8–42.0) (*n* = 30)	0.67
**Progression rate** (median, IQR) (1st to last visit)	1.3 (0.8–1.7) (*n* = 12)	1.3 (0.9–1.7) (*n* = 16)	0.34
**BMI** ^b^ **in kg/m²** (median, IQR)	24.9 (21.9–26.6) (*n* = 25)	24.2 (20.8–25.8) (*n* = 29)	0.51
**Diagnostic delay in months** (median, IQR)	9.0 (6.0–14.0) (*n* = 26)	8.0 (6.0–18.0) (*n* = 28)	0.93
**Survival in months** (median, HR, 95% CI)	61.0 (0.49, 0.19–1.29) (*n* = 27)	38.0 (2.03, 0.77–5.34) (*n* = 30)	0.12
**Neurofilaments in CSF in pg/ml** (median, IQR)	3135 (1080–6453) (*n* = 7)	2833 (1880–5885) (*n* = 9)	0.79
	***C9orf72* < 2000 repeats (*n* = 91)**	***C9orf72* > 2000 repeats (*n* = 74)**	***P* value**
**Age of onset** (median, IQR)	56.0 (50.0–63.0) (*n* = 76)	59.0 (54.0–65.0) (*n* = 65)	**0**.**01**
**Sex**			0.44
Male	56.0% (*n* = 51)	50.0% (*n* = 37)	
Female	44.0% (*n* = 40)	50.0% (*n* = 37)	
**Onset**			0.91
Spinal	71.0% (*n* = 44)	71.9% (*n* = 41)	
Bulbar	29.0% (*n* = 18)	28.1% (*n* = 16)	
**ALSFRS-R^a^** (1st visit) (median, IQR)	39.0 (35.0–43.0) (*n* = 73)	40.0 (35.0–42.0) (*n* = 59)	0.96
**Progression rate** (median, IQR) (1st to last visit)	1.3 (0.7–2.0) (*n* = 21)	1.1 (0.8–1.8) (*n* = 21)	0.71
**BMI^b^ in kg/m²** (median, IQR)	23.8 (21.5–26.2) (*n* = 58)	24.2 (21.2–26.2) (*n* = 60)	0.70
**Diagnostic delay in months** (median, IQR)	8.0 (5.0–14.0) (*n* = 71)	8.0 (3.0–17.0) (*n* = 55)	0.82
**Survival in months** (median, HR, 95% CI)	40.0 (0.98, 0.53–1.79) (*n* = 76)	38.0 (1.02, 0.56–1.88) (*n* = 63)	0.89
**Neurofilaments in CSF in pg/ml** (median, IQR)	3309 (2003–5796) (*n* = 14)	2833 (1538–5885) (*n* = 17)	0.99

Bold *P*-values are statistically significant.

a ALSFRS-R, Amyotrophic Lateral Sclerosis Functional Rating Scale-Revised; ^b^BMI, body mass index.

### Neuropsychological testing

Cognitive and behavioural function was evaluated by analysing data from ECAS in *n* = 106 patients (Table 3). Thirty-six patients had a positive family history for unclassified dementia, nine for parkinsonism, six for depression, three for M. Alzheimer, two for FTD and three for other unclassified psychiatric diseases. The median age at the time of first ECAS was 59.0 years (IQR 54.0–66.0 years) and 12.0 months (IQR 6.0–23.0 months) after disease onset.

**Table 3:** Neuropsychological characteristics in the Edinburgh Cognitive and Behavioural Screen (ECAS)

	*C9orf72* (*n* = 80)	Sporadic (*n* = 753)	*P* value	*SOD1* (*n* = 20)	*P*-value
**Non-**amyotrophic lateral sclerosis **specific score** (median, IQR)	27.5/36 (23.3–30.8) (*n* = 80)	28.0/36 (24.0–30.8) (*n* = 738)	0.83	29.0/36 (24.8–33.8) (*n* = 20)	0.14
**Memory** (median, IQR)	16.0/24 (12.3–19.0) (*n* = 80)	17.0/24 (13.0–19.0) (*n* = 738)	0.87	17.5/24 (15.3–21.8) (*n* = 20)	0.12
**Spatial perception** (median, IQR)	12.0/12 (11.0–12.0) (*n* = 80)	12.0/12 (11.0–12.0) (*n* = 742)	0.80	12.0/12 (12.0–12.0) (*n* = 20)	0.66
Amyotrophic lateral sclerosis**-specific score** (median, IQR)	76.0/100 (68.0–86.0) (*n* = 79)	80.0/100 (71.0–86.8) (*n* = 731)	0.09	87.0/100 (78.5–88.0) (*n* = 20)	**0**.**002**
**Verbal fluency** (median, IQR)	16.0/24 (12.0–18.0) (*n* = 80)	18.0/24 (14.0–20.0) (*n* = 736)	**0**.**004**	20.0/24 (18.0–22.0) (*n* = 20)	**<0**.**001**
**Language** (median, IQR)	25.0/28 (23.0–28.0) (*n* = 79)	26.0/28 (23.0–27.0) (*n* = 737)	0.13	26.5/28 (23.3–28.0) (*n* = 20)	0.51
**Executive functions** (median, IQR)	36.0/48 (31.3–41.0) (*n* = 80)	38.0/48 (32.0–41.0) (*n* = 734)	0.78	41.0/48 (37.3–42.0) (*n* = 20)	**0**.**01**
**Total score** (median, IQR)	102.0/136 (92.0–115.0) (*n* = 79)	107.0/136 (96.0–115.0) (*n* = 732)	0.54	115.0/136 (99.8–121.8) (*n* = 20)	**0**.**03**

Bold *P*-values are statistically significant.

Normal results were obtained for spatial perception (12/12, IQR 11.0–12.0) and language (25/28, IQR 23.0–28.0). Reduced scores were found for memory (16/24, IQR 12.3–19.0), verbal fluency (16/24, IQR 12.0–18.0) and executive functions (36/48, IQR 31.3–41.0). Due to deficits in the associated domains, both the amyotrophic lateral sclerosis-specific score (75/100, IQR 68.0–86.0) and the non-amyotrophic lateral sclerosis-specific score (27.5/36, IQR 23.3–30.8) were abnormal. Consequently, the total score (102/126, IQR 92.0–115.0) was also out of the normal range.

In relation to the comparator groups *SOD1* (*n* = 20) and sporadic amyotrophic lateral sclerosis (*n* = 753), *C9orf72* patients generally obtained the worst results in all domains (Fig. 1C**)** with the exception of spatial perception which was normal in all three groups. These differences were statistically significant for verbal fluency (*P* < 0.001), executive functions (*P* = 0.01), the amyotrophic lateral sclerosis-specific (*P* = 0.002) and total score (*P* = 0.03) compared to *SOD1*, and for verbal fluency (*P* = 0.004) compared to sporadic patients.

A follow-up ECAS performed after a median time interval of 12.5 months (IQR 8.0–15.5) was available in *n* = 16 patients. In this follow-up examination, total scores (106/136, IQR 85.5–113.8; *P* = 0.96), amyotrophic lateral sclerosis-specific scores (78/100, IQR 68.3–86.0; *P* = 0.70) and non-amyotrophic lateral sclerosis-specific scores (27/36, IQR 23.3–30.0; *P* = 0.27) were not significantly different compared to baseline. In the *C9orf72* group, 10 out of 66 rated patients (15.2%), were consistent with diagnosis of FTD in neuropsychological examination which was higher compared to sporadic amyotrophic lateral sclerosis (18/304 (5.9%); *P* = 0.02) and *SOD1* (0/11 (0%), *P* = 0.34).

## Discussion

To our knowledge, this study represents one of the largest number of cases and one of the most comprehensive analysis of clinical and genetic characteristics in amyotrophic lateral sclerosis patients with *C9orf72* mutations. Compared to two comparator groups of patients with *SOD1* mutations and sporadic patients, we identified multiple distinct clinical features in patients with *C9orf72* mutations.

Most surprisingly, we found that the majority of *C9orf72* patients had no evidence of a positive family history of amyotrophic lateral sclerosis. This finding is a major difference compared to *SOD1* patients who almost exclusively featured a positive family history for amyotrophic lateral sclerosis. One possible explanation would be that *C9orf72* mutations may cause various phenotypes including pure FTD which may make the identification of affected family members more difficult. Furthermore, in literature, other possible neurodegenerative disease entities associated with *C9orf72* repeat expansions have been discussed.^16^

Also, since *C9orf72*-associated amyotrophic lateral sclerosis generally affects older patients if compared to *SOD1,* and due to the steadily increasing life expectancy, it is possible that ancestors of actual *C9orf72* patients may not have reached the respective age to develop clinical signs of amyotrophic lateral sclerosis. In this study, the median age of onset in the *C9orf72* cohort was 58 years which is consistent with previous studies^17-21^ and may not seem very old at first glance. However, it should be noted that the average life expectancy for boys in Germany in the 1930s was only slightly higher. A study examining the incidence of *C9orf72* mutations in amyotrophic lateral sclerosis and FTD found that the pathogenic expansion of *C9orf72* hexanucleotides was non-penetrant in individuals younger than 35 years, 50% penetrant by 58 years and almost fully penetrant by 80 years.^22^ Furthermore, incomplete penetrance and anticipation effects must be considered. Our results are in line with a large Italian multicentre study, in which 55% of *C9orf72* patients had no indication of a positive family history.^18^ Comparable results were also found in a large Finish study which found 54% sporadic cases within the *C9orf72* cohort, although in this case, the specific genetic Finish background has to be considered.^20^ A large meta-analysis based on publications of *C9orf72* patients from 22 countries from Europe, North America, South America and Asia even classified 74% as ‘sporadic’.^23^ On the other hand, a French study found a vastly different proportion of only 8.6% sporadic patients.^19^ However, this apparent contradiction may be caused by a selection bias as the latter study specifically focused on families with multiple amyotrophic lateral sclerosis cases. Thus, we can conclude that the lack of a positive family history seems to be a common feature in patients with *C9orf72* mutations.

As opposed to sporadic amyotrophic lateral sclerosis, we found that the share of male and female patients was comparable in *C9orf72* which is consistent with previous reports.^17-20,24^ Likewise, the proportion of approximately two thirds of patients with spinal onset and one third of patients with bulbar onset in *C9orf72* is in line with pre-existing data.^18-20,25^ Therefore, we can conclude that the share of bulbar patients in *C9orf72* is higher compared to sporadic amyotrophic lateral sclerosis, and much higher than in *SOD1* patients who very rarely feature a bulbar onset. This phenomenon might be partly explained by the more frequent occurrence of bulbar forms in women and in older age. The age of onset in *C9orf72* was older compared to *SOD1*, but younger compared to sporadic amyotrophic lateral sclerosis. The multistep model can provide a possible explanatory approach. It describes the assumption that amyotrophic lateral sclerosis, similar to cancer, is triggered by the accumulation of multiple triggering risk factors.^26^ According to the model, in sporadic forms, six of such steps are necessary, whereas a mutation in the *C9orf72* gene requires only three steps, and a mutation in the *SOD1* gene only two.^27^ Since *C9orf72* mutation carriers require more environmental risk factors^28-30^ than *SOD1* mutation carriers, but less compared to non-mutation carriers, the disease might occur later compared to *SOD1* and earlier compared to sporadic patients.

Importantly, *C9orf72* patients featured a faster progression rate compared to *SOD1* patients and sporadic patients. In a study by Mandrioli *et al*., an even higher progression rate of 1.86 was found.^21^ Consistently, survival was also significantly reduced in *C9orf72* compared to *SOD1*. The finding of a faster disease progression in *C9orf72* patients is further corroborated by significantly higher p-NfH concentrations in the CSF in relation to the comparator groups. Regarding the comparison with *SOD1*, it is important to point out that European *SOD1* mutations (including the majority of mutations in this study) are known to be associated with a slow progression rate.^15^ Therefore, this result is not transferable to regions in which malign *SOD1* mutations are more prevalent, such as A4V mutations in the USA.

Interestingly, there were no significant associations between the clinical features of patients with *C9orf72* mutations and their hexanucleotide repeat lengths as tested with various thresholds. Thus, repeat lengths apparently do not appear to constitute a decisive factor for the severity of the disease. On the contrary, younger age of onset was even associated with shorter repeat lengths. Whether this might be a random finding must be assessed by future studies with even larger numbers of cases. One possible explanation could be that not the repeat length, but rather the methylation status constitutes the decisive factor for the age of onset of the disease. In accordance with this hypothesis, it has been shown previously that not the methylation level at any single CpG site, but rather an acceleration of DNA methylation age (DNA methylation age minus chronological age) in general is correlated with an earlier age of onset and shorter disease duration.^31^ On the other hand, by analysing and comparing blood samples of *C9orf72* amyotrophic lateral sclerosis and FTD patients, one study found that *C9orf72* hypermethylation was associated with later age at death and longer disease duration in FTD, but not in amyotrophic lateral sclerosis.^32^ Since the number of GGGGCC hexanucleotide repeats and thus also the frequency of amyotrophic lateral sclerosis caused by *C9orf72* mutations vary depending on the genetic background, it would be interesting to compare the clinical features in other populations.

Neuropsychological examinations in *C9orf72* patients revealed reduced verbal fluency, memory and executive functions. This is in line with previous publications which found that these cognitive functions were predominantly affected in the general amyotrophic lateral sclerosis population.^33^ Thus, we did not detect a *C9orf72*-specific pattern of neuropsychological deficits. However, the ECAS results of *C9orf72* patients turned out to be worse in every domain, with the exception of spatial perception, in relation to the groups of patients with *SOD1* and sporadic patients. Therefore, it can be concluded that neuropsychological deficits are more accentuated in *C9orf72*, although, compared to sporadic amyotrophic lateral sclerosis, this finding was only significant for verbal fluency. Of note, reduced verbal fluency, non-verbal memory and executive functions were also found in presymptomatic *C9orf72* gene carriers.^34^ Behavioural abnormalities suspicious for FTD were found in 15.2% of rated *C9orf72* cases which is not surprising as *C9orf72* mutations can cause both amyotrophic lateral sclerosis and FTD.^3,9^ Our results are in line with the findings of Mandrioli *et al*. who reported with 10.7% a higher share of FTD in *C9orf72* amyotrophic lateral sclerosis patients compared to amyotrophic lateral sclerosis patients without genetic mutations.^21^

Our study is not without limitations. Retrospective analysis and multicentre data collection may result in an unequal representation of clinical features as well as differences regarding the number and interval of follow-up visits. Furthermore, the sole participation of tertiary centres might imply a selection bias. However, at the same time, thorough clinical phenotyping performed by physicians experienced in the diagnosis of amyotrophic lateral sclerosis can also be considered as strengths. Losses to follow-up limit more detailed analysis of survival data. The significance of neuropsychological data is limited by the low number of follow-up examinations and the restriction to ECAS. The determination of repeat lengths by Southern blot depends on technical and investigator-dependent factors, which may influence the accuracy according to previous publications.^35^ Also, it must be considered whether the determination of repeat lengths in blood samples is suitable for deriving associations with clinical features and disease courses, or whether brain tissue must be examined due to the assumption of somatic mosicism.^35,36^

In summary, the findings of this study show that clinical characteristics of amyotrophic lateral sclerosis patients with *C9orf72* mutations differ significantly from sporadic amyotrophic lateral sclerosis patients and *SOD1* gene carriers, including a higher share of bulbar onset and female patients, faster disease progression rates, higher neurofilament levels in CSF, a larger percentage of neuropsychological deficits and shorter survival. We also found a surprisingly high number of patients without positive family histories. The careful clinical phenotyping of patients with specific genetic mutations will help to facilitate earlier diagnosis, provide selection criteria for genetic testing and define a comparator standard for future studies on *C9orf72*.

## Supplementary Material

fcad087_Supplementary_Data

## Abbreviations

ALSFRS-R =: Amyotrophic Lateral Sclerosis Functional Rating Scale-Revised
BMI =: body mass index
ECAS =: Edinburgh Cognitive and Behavioural Scale
FTD =: frontotemporal dementia
p-NfH =: phosphorylated neurofilament heavy chain
