# Supplementary material for: Clinical and genetic features of amyotrophic lateral sclerosis patients with C9orf72 mutations
Source: Brain Commun. 2023 Mar 21;5(2):fcad087. doi: 10.1093/braincomms/fcad087 (PMC10065188; doi:10.1093/braincomms/fcad087)
Supplement: fcad087_Supplementary_Data [file fcad087_supplementary_data.docx]

**Supplementary table 1:** *SOD1* mutation spectrum

| **Mutation** | Number of patients |
| --- | --- |
| **p.Glu41Gly** | 1 |
| **p.His44Arg** | 2 |
| **p.His47Arg** | 2 |
| **p.Leu85Phe** | 1 |
| **p.Asn87Ser** | 1 |
| **p.Ile114Thr** | 1 |
| **p.Gly148Asp** | 1 |
| **p.His49Arg** | 2 |
| **p.Gly73Ser** | 3 |
| **p.Val88Ala** | 2 |
| **p.Asp91Ala** | 11 |
| **p.Glu101Lys** | 5 |
| **p.Ile105Phe** | 2 |
| **p.Ile113Thr** | 2 |
| **p.Arg116Gly** | 26 |
| **p.Leu145Phe** | 6 |
| **p.Val149Gly** | 2 |
